# Variations in hospital resource use across stroke care teams in England, Wales and Northern Ireland: a retrospective observational study

**DOI:** 10.1136/bmjopen-2019-030426

**Published:** 2019-09-20

**Authors:** David G Lugo-Palacios, Brenda Gannon, Matthew Gittins, Andy Vail, Audrey Bowen, Sarah Tyson

**Affiliations:** 1 Manchester Centre for Health Economics, University of Manchester, Manchester, UK; 2 School of Economics & Centre for Business and Economics of Health, Faculty of Business, Economics and Law, The University of Queensland, Australia; 3 Centre for Biostatistics, Manchester Academic Health Science Centre, University of Manchester, Manchester, UK; 4 Division of Neuroscience & Experimental Psychology, Manchester Academic Health Science Centre, University of Manchester, Manchester, UK; 5 Division of Nursing, Midwifery and Social Work, Manchester Academic Health Science Centre, University of Manchester, Manchester, UK

**Keywords:** length of stay, performance measurement, resource use, SSNAP, stroke

## Abstract

**Objective:**

To identify the main drivers of inpatient stroke care resource use, estimate the influence of stroke teams on the length of stay (LoS) of its patients and analyse the variation in relative performance across teams.

**Design:**

For each of four types of stroke care teams, a two-level count data model describing the variation in LoS and identifying the team influence on LoS purged of patient and treatment characteristics was estimated. Each team effect was interpreted as a measure of stroke care relative performance and its variation was analysed.

**Setting:**

This study used data from 145 396 admissions in 256 inpatient stroke care teams between June 2013 and July 2015 included in the national stroke register of England, Wales and Northern Ireland—Sentinel Stroke National Audit Programme.

**Results:**

The main driver of LoS, and thus resource use, was the need for stroke therapy even after stroke severity was taken into account. Conditional on needing the therapy in question, an increase in the average amount of therapy received per inpatient day was associated with shorter LoS. Important variations in stroke care performance were found within each team category.

**Conclusions:**

Resource use was strongly associated with stroke severity, the need for therapy and the amount of therapy received. The variations in stroke care performance were not explained by measurable patient or team characteristics. Further operational and financial analyses are needed to unmask the causes of this unexplained variation.

**Strengths and limitations of this study**

- Analyses a dataset from the Sentinel Stroke National Audit Programme (SSNAP) which is the single source of stroke data in England, Wales and Northern Ireland and contains records from 95% of all patients admitted to hospital with acute stroke.
- Given that the care provided and the way teams are organised differ depending on their category, this study performs separate analysis for each of the four categories in which stroke teams are classified in the UK.
- Identifies variation in resource use that is not explained by patient and treatment characteristics and interprets it as performance measure.
- Does not analyse costs variation across stroke teams since SSNAP does not provide cost information.
- It is a partial resource use analysis of stroke care as SSNAP only reports data on stroke hospital admissions; further hospital admissions with different primary diagnosis, as well as outpatient specialist and general practice (GP) visits are not included in the analysis.

## Introduction

Stroke is a leading cause of mortality and disability worldwide.^1^ In the UK there are more than 100 000 strokes per year; more than 80% of whom survive their stay in hospital, but two thirds are left with a long-term disability.^2^ The prevalence, short-term and long-term consequences of stroke are reflected in the high use of resources associated with stroke care. Previous research estimated that the aggregated cost of stroke to UK society is £26 billion a year and that informal care contributes with approximately £15.8 billion which is almost double the National Health Service (NHS) and the Personal Social Service stroke care costs.^3^

The important health and financial burdens of stroke have caught the attention of UK policymakers in recent years. The Department of Health’s National Stroke Strategy for England recommended major changes in the delivery of stroke and identified that care in a stroke unit was the single biggest factor that can improve outcomes after stroke.^4 5^ Consequently, in an effort to improve the quality and efficiency of stroke care, every hospital in England, Wales and Northern Ireland that routinely admits patients who had a stroke now has a specialist stroke unit and, moreover, acute stroke services were centralised in London and Greater Manchester in 2010.^6–8^

Recent studies have focused on the study of the effect of centralising acute stroke care, but resource use within both acute and postacute stroke units across the UK has received little attention in the literature.^4 6 7 9^

Hospital resource use has recently been studied by analysing the variation of patient-level costs and/or inpatient length of stay (LoS) for a particular type of care across hospitals.^10 11^ Hospital cost data at the patient level are not always available, but LoS is often used as a proxy for costs since LoS data can be obtained straightforwardly from administrative data.^10–13^ In fact, for some European countries, it has been shown that there is a strong correlation between stroke care costs and inpatient LoS.^13^ Furthermore, analysis based on LoS rather than costs may prove more powerful at fostering behaviour change as clinicians have more direct influence on LoS than on costs.^10^

LoS for a particular type of care may vary among patients because they have very different characteristics or are diagnosed and treated differently, and also due to the characteristics of the hospital where their treatment is delivered, both within and beyond the control of hospital managers.^11^ Reductions in the LoS required for a particular type of care can reduce the costs of undertaking a fixed number of hospitalisations and increase the amount of work that hospitals can undertake within their fixed budget.^11 12 14^ Therefore, after conditioning on patient and treatment characteristics, several studies have identified the relative influence that each hospital has on its patients’ LoS, interpreted this influence as a measure of hospital performance and explored hospital-level characteristics that could explain differences across hospitals.^10 12 15–18^ The present paper builds on this literature by analysing variations in inpatient resource use and stroke care performance within different classifications of stroke care teams across the UK. In particular, the study presented here identifies the main drivers of inpatient stroke care resource use, estimates the influence of each stroke care team on its patients’ LoS over and above patient and treatment characteristics and analyses the variation in relative performance across stroke care teams.

## Methods

### Data sources

This study uses patient-level data from the national stroke register of England, Wales and Northern Ireland—Sentinel Stroke National Audit Programme (SSNAP)—from June 2013 to July 2015.^19^ SSNAP is a unique dataset as it is the single source of stroke data in these countries; it contains records from 95% of all patients admitted to hospital with acute stroke, including information about the care received throughout the stroke care pathway, length of stay in each part of the stroke service and the different types of therapy received by each patient.^20 21^ SSNAP also provides data on the structure of stroke services measuring, for example, the size of the stroke care workforce and the kind of rehabilitation services available at stroke units.^22^

A stroke unit is a ward in a hospital where patients who had a stroke are cared for by a multidisciplinary team of professionals who specialise in stroke care. The team usually consists of doctors, nurses, physiotherapists, occupational therapists, speech and language therapists, dietitians, therapy assistants, social workers and, sometimes, psychologists.^8^

SSNAP classifies inpatient stroke care teams in three categories: Routinely Admitting Teams (RATs), Non-Routinely admitting Acute Teams (NRATs, these are typically a stroke units which takes patients transferred from hyper-acute care and provides acute care and rehabilitation) and Non-Acute Inpatient Teams (NAITs, typically a specialist stroke rehabilitation unit).^23^ RATs can also be categorised as those specifically providing hyper-acute care (rapid assessment with specialist clinical teams, diagnostic facilities and the capacity to provide thrombolysis) for the first 72 hours after stroke and those which provide acute stroke care (with or without thrombolysis) over the first 7 days after stroke. At the time of this project, specific hyper-acute stroke units (HASUs) were operating in London and Greater Manchester.^4^ Further details on the distribution of type of stroke teams across the country are available from the SSNAP website.^23^ Since the care provided and the way stroke teams are organised differ depending on their category, it was decided to conduct separate analyses for each of the four categories. However, the data from the Greater Manchester HASUs could not be distinguished from other types of stroke unit also operating in the same locations. Therefore, 4485 observations from these hospitals were excluded from the analysis.

The main outcome variable of this study is the LoS in the inpatient stroke team defined as ‘the time from admission to stroke team in question until transfer to another stroke team or hospital discharge’. This means that it was possible to distinguish the LoS from patients originally admitted to a RAT or HASU and then transferred to another stroke team (NRAT or NAIT) within the same location/site/hospital and analyse them separately. LoS in SSNAP is recorded in minutes; however for ease of interpretation, we have expressed LoS in full days by transforming the SSNAP record into days and rounding it to the closest integer.

LoS distributions are highly skewed and this may influence estimation of the relative influence that each hospital has on the LoS of its patients. For this reason, right-tailed LoS outliers were excluded from the econometrics analyses. Outliers were identified for each category of inpatient stroke teams with a threshold based on three times the standard deviation (SD) of the LoS distribution for each type of team category.^10^ The cut-off point to define outliers for each case is calculated by first computing the number of days exceeding the national average LoS by three SD and then rounding this number to the next complete month (30 days per month). For example, for NRATs, the three SD threshold is 113 days, hence the cut-off point is rounded to 120 days to match four complete months. An observation is classified as an outlier if its LoS exceeds 30 days for HASUs, 90 days for RATs, 120 days for NRATs and 150 days for NAITs. This resulted in 2410 admissions being identified as LoS outliers and dropped from the final sample.

After receiving acute care, some stroke patients need further rehabilitation in the community. Early supported discharge (ESD) is a system in which rehabilitation is provided to stroke patients at home instead of at hospital.^22^ Care provided by ESD teams is also reported in SSNAP. However, since the focus of this study is on hospital-based care ~20 000 observations describing community-based care are not included in the analysis. Likewise, observations from stroke units with fewer than 24 admissions—one per month—during the study period were dropped from the analysis (282 admissions). Additional exclusion criteria were admissions with no stroke symptoms (ie, National Institutes of Health Stroke Scale (NIHSS) score of zero), errors in the LoS data (23 cases when LoS in stroke unit was longer than the overall inpatient LoS); 774 observations where LoS in the stroke team could not be recovered from SSNAP; and missing values in any of the admission-specific covariates. The final sample used in this analysis includes 145 396 admissions from 256 stroke care teams.

### Patient and public involvement (PPI)

Members of the Patient and Public Involvement panel of the University of Manchester’s Stroke Research Centre have contributed throughout the project. The panel consists of stroke survivors and their families/carers who provide a PPI perspective for stroke research in Manchester. It was founded by the NW Stroke Research Network and continued by the University of Manchester Stroke Research Group after the stroke research network’s demise. It is led by a stroke survivor. The panel has 30+ members of all ages, types and severity. The panel supported the project, highlighting that difficulty accessing appropriate therapy to meet their needs was a cause of great concern for many stroke survivors and a major cause of dissatisfaction with stroke services.

### Analysis

Following the two-step approach suggested by Street *et al*, Gaughan *et al* and Laudicella *et al* this study uses the LoS of patients in stroke under the supervision of stroke care teams (as a proxy for health care costs) to analyse variations in resource use and stroke care performance across England, Wales and Northern Ireland.^10 12 24^

The first stage specifies a multilevel model which considers that stroke admissions (level 1) are clustered within stroke teams (level 2) to estimate the team influence on LoS purged of patient and admission-specific characteristics. Each team effect can be interpreted as a measure of relative performance with higher values implying that a team uses more resources than other teams to treat patients who had a stroke.^25 26^

LoS is considered count data as it tends to take a limited set of low values with many patients having short LoS and relatively few staying for longer periods.^11^ The Poisson and the negative binomial (NB) regression models are the most used techniques to analyse count data. NB distributions are often used to model overdispersed data (variance higher than mean) as the Poisson model yields biased estimates in the presence of overdispersion.^27^ As shown in table 1, LoS in SSNAP is overdispersed and, therefore, this model is used to describe the variation in this variable.

**Table 1:** Descriptive statistics

Variable	HASUs*	RATs†	NRATs	NAITs
No. of patients	14 720	112 339	11 693	6644
No. of stroke teams	8	147	32	66
Percentages of patients (%)				
Age				
<50 years	8.47	5.12	5.69	3.49
50–59 years	11.84	8.40	8.76	6.73
60–69 years	16.55	16.04	13.72	14.83
70–79 years	25.39	26.77	25.12	26.78
80–89 years	28.31	32.16	33.98	36.14
90–99 years	9.08	11.21	12.30	11.86
>100 years	0.35	0.30	0.44	0.18
Gender				
Male	51.78	49.68	48.13	48.43
Ethnicity				
White	62.35	92.60	67.48	91.59
Asian	9.99	1.91	8.71	2.23
Black	7.47	0.54	7.12	0.84
Mixed	0.92	0.21	0.79	0.18
Ethnicity not known	12.11	4.32	10.31	4.29
Ethnicity other	7.16	0.42	5.58	0.87
Social-economic deprivation				
Low deprivation	31.51	21.36	30.63	16.68
Moderate deprivation	29.91	22.79	28.48	24.89
High deprivation	20.62	24.62	20.15	25.81
Very high deprivation	14.94	23.24	15.18	20.14
Deprivation not known	3.02	8.00	5.56	12.48
Stroke related co-morbidities				
Heart failure	6.28	5.52	7.09	5.10
Hypertension	61.83	53.57	62.46	54.21
Atrial fibrillation	17.87	21.12	21.64	21.73
Diabetes	24.00	19.34	23.67	19.37
Previous stroke	24.21	27.55	26.05	25.98
Disability				
Previous mRS=1	–	–	6.69	6.44
Previous mRS=2	–	–	12.13	9.47
Previous mRS=3	–	–	18.85	21.22
Previous mRS=4	–	–	40.54	46.64
Previous mRS=5	–	–	21.80	16.23
Stroke severity				
Mild stroke (NIHSS<5)	44.35	45.22	30.43	27.26
Moderate stroke (NIHSS 5–14)	35.94	33.85	43.60	46.81
Severe stroke (NIHSS 15–20)	9.71	9.43	13.67	13.76
Very severe (NIHSS>20)	10.00	11.50	12.31	12.18
Stroke pathology				
Intracerebral haemorrhage	11.40	10.70	13.15	13.97
Need for therapy				
Occupational therapy (n_OT)	79.93	79.78	87.35	95.15
Physiotherapy (n_PT)	83.13	84.58	88.94	95.89
Speech and language therapy (n_SALT)	56.11	45.25	65.39	62.73
Psychological therapy (n_PSY)	2.85	3.32	14.87	18.08
Therapy service model				
Therapy available at weekends	25.82	25.43	25.80	25.93
Patient transferred from a HASU	0.10	0.10	67.23	0.98
Adverse events				
Patient suffered a urinary tract infection	6.17	4.76	8.18	8.19
Mortality				
Deceased	4.97	14.79	12.01	4.38
Transferred to another inpatient unit	55.56	10.22	18.40	4.82
Mean (SD)				
LoS‡	4.10 (3.67)	13.93 (17.39)	23.14 (23.90)	37.37 (26.36)
n_OT x OT average daily minutes	19.95 (19.05)	15.53 (17.17)	17.85 (17.28)	17.65 (14.94)
n_PT x PT average daily minutes	20.52 (16.68)	15.29 (14.28)	18.28 (15.97)	19.46 (13.98)
n_SALT x SALT average daily minutes	10.63 (14.92)	4.60 (8.65)	7.61 (10.79)	6.00 (9.51)
n_PSY x PSY average daily minutes	0.54 (3.86)	0.10 (0.92)	0.88 (4.06)	0.55 (1.80)
Order of team in patient pathway	1.01 (0.13)	1.03 (0.18)	2.00 (0.47)	2.11 (0.48)

*Units in Greater Manchester that have HASUs are not considered here.

†HASUs are essentially RATs, the descriptive statistics reported here for RATs exclude those that are also classified as HASUs.

‡SSNAP records LoS in minutes. To ease interpretation, this record is reported in days by dividing SSNAP record by 1440 and rounding it to the closest integer number.

HASUs, hyper-acute stroke units; LoS, length of stay; mRS, modified Rankin Scale; NAITs, Non-Acute Inpatient Teams; NIHSS, National Institutes of Health Stroke Scale; NRATs, Non-Routinely admitting Acute Teams; RATS, Routinely Admitting Teams; SSNAP, Sentinel Stroke National Audit Programme.

The associated probability of observing the count *y_ik_* (ie, LoS in number of days) in the most common version of the NB model, is

$$f(y_{ik}|X_{ik},h_{k})=\frac{\Gamma (\propto^{-1}+y_{ik})}{\Gamma (\propto^{-1})\Gamma (y_{ik}+1)}{\left(\frac{\propto^{-1}}{\lambda_{ik}+\propto^{-1}}\right)}^{\propto^{-1}}{\left(\frac{\lambda_{ik}}{\lambda_{ik}+\propto^{-1}}\right)}^{y_{ik}}$$

where Γ(.) is the gamma function. The first two conditional moments are

$$E\left[y_{ik}|X_{ik}\right]=\lambda_{ik}=\exp\left(X_{ik}\beta +u_{k}h_{k}\right)$$

$$V\left[y_{ik}|X_{ik},h_{k}\right]=\lambda_{ik}+\propto^{\prime }\lambda_{ik}^{2},\alpha >0$$

Where ∝ is a constant overdispersion parameter to be estimated with the rest of the parameters. $y_{ik}$ is the LoS (number of days) of patient *i* in team *k*; $X_{ik}$ is a vector of patient and treatment characteristics, used as proxy for case-mix; $h_{k}$ represents the deviation of team *k* from the grand mean. $u_{k}$ is, therefore, the estimated team effect on LoS (performance measure), which is analogous to a fixed effect in linear models.^28^

The $X_{ik}$ vector includes demographic characteristics such as the patient’s age group; a dummy variable indicating whether the patient was a male; and five dummy variables identifying the ethnicity of the patient. $X_{ik}$ also captures the patient’s social deprivation quartile and whether he/she suffered a previous stroke, congestive heart failure, hypertension, atrial fibrillation and/or diabetes prior to his/her current stroke admission. While the use of validated comorbidity measures (eg, Elixhauser or Charlson) could be preferred to the inclusion of the latter indicator variables, SSNAP does not record secondary diagnoses and, thus, it was not possible to build any of the commonly used comorbidity indexes. Stroke severity is taken into account by including in $X_{ik}$ the following factors: three dummy variables indicating whether the stroke was moderate, severe or very severe, taking mild as reference, according to the NIHSS score on admission; a binary variable that identifies whether the stroke is a primary intracerebral haemorrhage (or ischaemic stroke); four dummy variables each indicating whether the patient required occupational therapy, physiotherapy, speech and language therapy and/or psychological therapy in each stay; and, the modified Rankin Scale (mRS, a measure of disability) score prior to the arrival at the stroke team in question expressed as a categorical variable taking the value of 2—slight disability—as reference. The latter is defined as the mRS at discharge of the previous team if the patient was transferred in from another stroke team. HASUs and RATs were the starting point in the care pathway of 99.3% and 97.9% of the patients hospitalised in these units, respectively. Therefore, the mRS score prior to arrival to the stroke team in question is not included in the model for HASUs and RATs. Stroke teams report the need for therapy in SSNAP by indicating, for each patient, whether he/she needed physiotherapy (PT), occupational therapy (OT), speech and language therapy (SALT) or psychological therapy (PSY) at any point during his/her stay in the stroke team.

In addition to patient characteristics, $X_{ik}$ also includes treatment factors such as the interaction between the need for each therapy and the average amount of therapy provided (in minutes) to the patient per inpatient day. The order of the stroke team in the patient’s pathway, as well as if the patient was first admitted during the weekend, and whether he/she was transferred from a HASU were also considered. To capture the presence of adverse events, often used as a proxy for inpatient care quality, a dummy variable indicates whether the patient developed a urinary tract infection in the first seven days following initial admission.^12^ Finally, a variable indicating whether the patient died during his/her stay at the stroke team was also included.

In the second stage, the variation in the estimated stroke team effects is analysed. Thus, following Street *et al*, variables measuring team-level and hospital-level characteristics are used in the following generalised least squares model with weights proportional to the inverse of the squared standard errors and Efron robust standard errors to correct for potential heteroscedasticity.^10 29^

$$\hat{u_{k}}=\gamma_{0}+\gamma^{\prime }z_{k}+\epsilon_{k}$$

The number of patients treated by the stroke care team (in hundreds) is one of the explanatory variables included in $z_{k}$ to investigate if economies of scale are associated with stroke care performance (ie, whether volume increases are associated with decreasing average costs, using LoS as proxy for costs). The mortality rate per 100 patients that each team observes is also included in $z_{k}$ to explore whether stroke teams with high mortality tend to have shorter average LoS. Additionally, the rate per 100 patients that left the stroke unit in a dependent status, measured using their mRS at discharge (dependent if mRS at discharge > 2), is included to examine the extent to which systematically discharging patients in this condition is related with the team’s performance measure. The rate of urinary infections per 100 patients is included as a covariate to study the association between quality of care and the measure of stroke care performance. The numbers of whole time equivalent professionals in clinical psychology, dietetics, occupational therapy, physiotherapy, speech and language therapy, and nursing per 100 admissions were are also included as covariates in the second stage. Dummy variables indicating whether thrombolysis is provided on site and whether the team has access to a specialist ESD, to a non-specialist ESD and to a non-specialist community rehabilitation team were also included in $z_{k}$. Finally, the geographical region where the team is based is also considered.

The second stage analysis was only conducted for RATs and NRATs as data for only eight HASUs were available and team-level data for NAITs were not always available.

## Results

Table 1 reports the descriptive statistics for the patient level data. 87% of total stays at specialised stroke care teams were recorded in routinely admitting stroke teams (including HASUs). Most admissions involved individuals aged 80–89 years old, followed by patients in the 70–79 age group. Most patients (>70% of stroke hospitalisations in all stroke team categories) had a mild or moderate stroke. A higher proportion of patients died while receiving care in a RAT (14.8%) than in a NAIT (4.38%).

Table 1 also shows that at least 80% of patients in all stroke care team categories required PT and/or OT while SALT was needed in at least 45% of the cases. Recorded need for PSY in HASUs and RATs is much lower (2.9% and 3.3%, respectively) than those in NRATs and NAITs (14.9% and 18%). Patients requiring therapy at some point during their stay in a HASU received on average 19.95 min of OT per inpatient day, 20.52 of PT, 10.63 of SALT and 0.54 of PSY. In RATs, the average minutes of therapy received per inpatient day are 15.53 for OT, 15.29 for PT, 4.60 for SALT and 0.10 for PSY. In NRATs, averages are 17.85 for OT, 18.28 for PT, 7.61 for SALT and 0.88 for PSY. Finally, in NAITs the average minutes per inpatient day are 17.65 for OT, 19.46 for PT, 6 for SALT and 0.55 for PSY.

Average LoS in HASUs, RATs, NRATs and NAITs is 4.10, 13.93, 23.14 and 37.37 days, respectively, and is always overdispersed in all team categories. While there are some observations with LoS=0 (discharged within the first 24 hours of admission to the stroke team in question), the percentage of these is 2% or lower. Therefore, no adjustment was needed to accommodate values equal to zero.

Results from the negative binomial models estimated in the first stage are reported in table 2 (and online supplementary table S1—see online version). In general, after conditioning for demographic characteristics, the estimated associations between LoS and the variables used as proxy for stroke severity have the same (positive) direction, but different magnitude across team categories. These associations are higher in RATs than in the rest of teams. For example, patients with moderate strokes, compared with those suffering mild ones, are, on average, hospitalised for 13% more days in HASUs, 18% in NAITS, 21% in NRATs and 53% in RATs. The magnitude of these associations increases noticeably within each team category for patients with severe and very severe strokes. In particular, patients admitted in NRATs for a very severe stroke stay on average 84% longer than those admitted for a mild stroke. Patients with intracerebral haemorrhage are also significantly associated with longer inpatient stays in HASUs, RATs and NRATs.

10.1136/bmjopen-2019-030426.supp1 Supplementary file 1

**Table 2:** Results Stage 1: negative binomial models for length of stay per team category

	HASUs (1)	RATs (2)	NRATs (3)	NAITs (4)
Previous mRS score, ref. group: mRS=2				
Previous mRS=0 or previous mRS=1	–	–	1.012	1.096*
	–	–	0.043	0.052
Previous mRS=3	–	–	1.145***	1.121***
	–	–	0.038	0.040
Previous mRS=4	–	–	1.467***	1.337***
	–	–	0.047	0.043
Previous mRS=5	–	–	1.655***	1.620***
	–	–	0.061	0.059
Severity of stroke, ref. group: mild (NIHSS<5)				
Moderate (NIHSS 5–14)	1.132***	1.533***	1.206***	1.184***
	0.015	0.012	0.026	0.024
Severe (NIHSS 15–20)	1.189***	1.823***	1.402***	1.276***
	0.026	0.021	0.041	0.033
Very severe (NIHSS>20)	1.221***	1.838***	1.427***	1.292***
	0.029	0.022	0.047	0.037
Intracerebral haemorrhage	1.081***	1.195***	1.098***	1.032
	0.022	0.012	0.025	0.022
Need for therapy				
Need for occupational therapy (n_OT)	1.779***	2.306***	2.083***	1.695***
	0.040	0.029	0.078	0.114
Need for physiotherapy (n_PT)	1.762***	1.754***	1.720***	1.691***
	0.046	0.025	0.075	0.115
Need for speech and language therapy (n_SALT)	1.456***	2.121***	1.778***	1.336***
	0.025	0.019	0.040	0.026
Need for psychological therapy (n_PSY)	1.613***	2.315***	1.920***	1.395***
	0.066	0.038	0.049	0.037
Amount of therapy				
n_OT x OT average daily minutes	0.985***	0.977***	0.986***	0.986***
	0.000	0.000	0.001	0.001
n_PT x PT average daily minutes	0.988***	0.995***	0.996***	1.001
	0.001	0.000	0.001	0.001
n_SALT x SALT average daily minutes	0.986***	0.970***	0.983***	0.994***
	0.001	0.001	0.001	0.001
n_PSY x PSY average daily minutes	0.986***	0.951***	0.973***	0.977***
	0.002	0.004	0.003	0.006
Order of team in patient pathway	–	1.381***	1.057***	1.059*
	–	0.027	0.020	0.032
Transferred from a HASU to another inpatient stroke unit	1.354	0.944	0.816***	0.758**
	0.262	0.071	0.020	0.086
Deceased	1.418***	0.830***	0.810***	0.765***
	0.049	0.009	0.024	0.044
Transferred to another inpatient stroke unit	1.197***	1.070***	0.661***	0.670***
	0.017	0.011	0.016	0.031
Constant	1.828***	2.512***	4.376***	8.768***
	0.051	0.066	0.291	0.940
N	14 720	112 339	11 693	6644
Alpha (dispersion)	0.128***	0.582***	0.494***	0.338***
Adj. Deviance R^2	0.452	0.481	0.471	0.331

Exponentiated coefficients (IRR); Standard errors in second row.

Team deviations not shown. *p<0.10, **p<0.05, ***p<0.01. Additional covariates not included in the table for presentation purposes: age, sex, ethnicity, social deprivation and stroke related co-morbidities.

HASUs, hyper-acute stroke units; mRS, modified Rankin Scale; NAITs, Non-Acute Inpatient Teams; NIHSS, National Institutes of Health Stroke Scale; NRATs, Non-Routinely admitting Acute Teams; NB, negative binomial; RATs, Routinely Admitting Teams.

The need for all types of therapy considered in this study is strongly associated with longer LoS; this result is consistent across all types of stroke teams. For instance, needing OT is associated with 131% longer stays in RATs and with 70%, 78% and 108% longer LoS in NAITs, HASUs and NRATs, respectively. The need for PT is associated with 76% longer LoS in HASUs, 75% in RATs, 72% in NRATs and 69% in NAITs. The range of the association of the need of SALT with longer LoS ranges between 34% in NAITs and 112% in RATs. Finally, patients needing PSY at some point of their stay in RATs have 132% longer LoS than patients in RATs who did not need this kind of therapy; this figure is 92% for patients in NRATs, 61% for patients in HASUs and 40% for patients in NAITs.

Conditional on needing the therapy in question, the daily average dose of therapy received across all team categories is associated with a shorter LoS. Moreover, the magnitude of each of the estimated associations is similar across HASUs, RATs, NRATs and NAITs. For example, an additional minute in the average of daily OT received is associated with a 1.5% shorter LoS in HASUs, a 2.3% shorter in RATs and a 1.4% shorter in NRATs and NAITs. The strongest negative association found was the one between LoS and the amount of PSY received per inpatient day in RATs as an additional minute in this average is associated with a 4.9% shorter stay. Table 3 reports the average marginal effects of increasing by 1 min the average of therapy provided per inpatient day for each type of therapy. This allows expressing the results of the NB models in days. For example, all else unchanged, an additional minute in the average of OT received per inpatient day by a patient in a RAT is associated with a 0.30 days (432 min=7.2 hours) shorter LoS; the respective numbers are 90 min for PT and PSY and 432 min for SALT.

**Table 3:** Average Marginal Effects of average daily minutes of therapy received, days of inpatient stay†

	HASUs	RATs	NRATS	NAITS
OT daily average	−0.052***	−0.300***	−0.313***	−0.533***
	0.002	0.004	0.016	0.048
PT daily average	−0.046***	−0.063***	−0.101***	0.020
	0.002	0.005	0.018	0.038
SALT daily average	−0.038***	−0.300***	−0.321***	−0.165***
	0.002	0.007	0.021	0.030
PSY daily average	−0.002***	−0.062***	−0.157***	−0.211***
	0.000	0.005	0.016	0.053

*p<0.10, **p<0.05, ***p<0.01.

†Conditional on needing the therapy in question. Standard errors in second row.

HASUs, hyper-acute stroke units; NAITs, Non-Acute Inpatient Teams; NRATs, Non-Routinely admitting Acute Teams; OT, occupational therapy; PT, physiotherapy; PSY, psychological therapy; RATs, Routinely Admitting Teams; SALT, speech and language therapy.

Among other significant results, it is important to highlight the following. Dying in hospital is associated with a 42% longer LoS in HASUs, but with a significantly shorter LoS in the other team categories. Patients transferred to another inpatient team (compared with those who are discharged without further transfers to another team) are associated with longer LoS in HASUs and RATs, but with a shorter stay in NRATs and NAITs.

**Figure 1:**
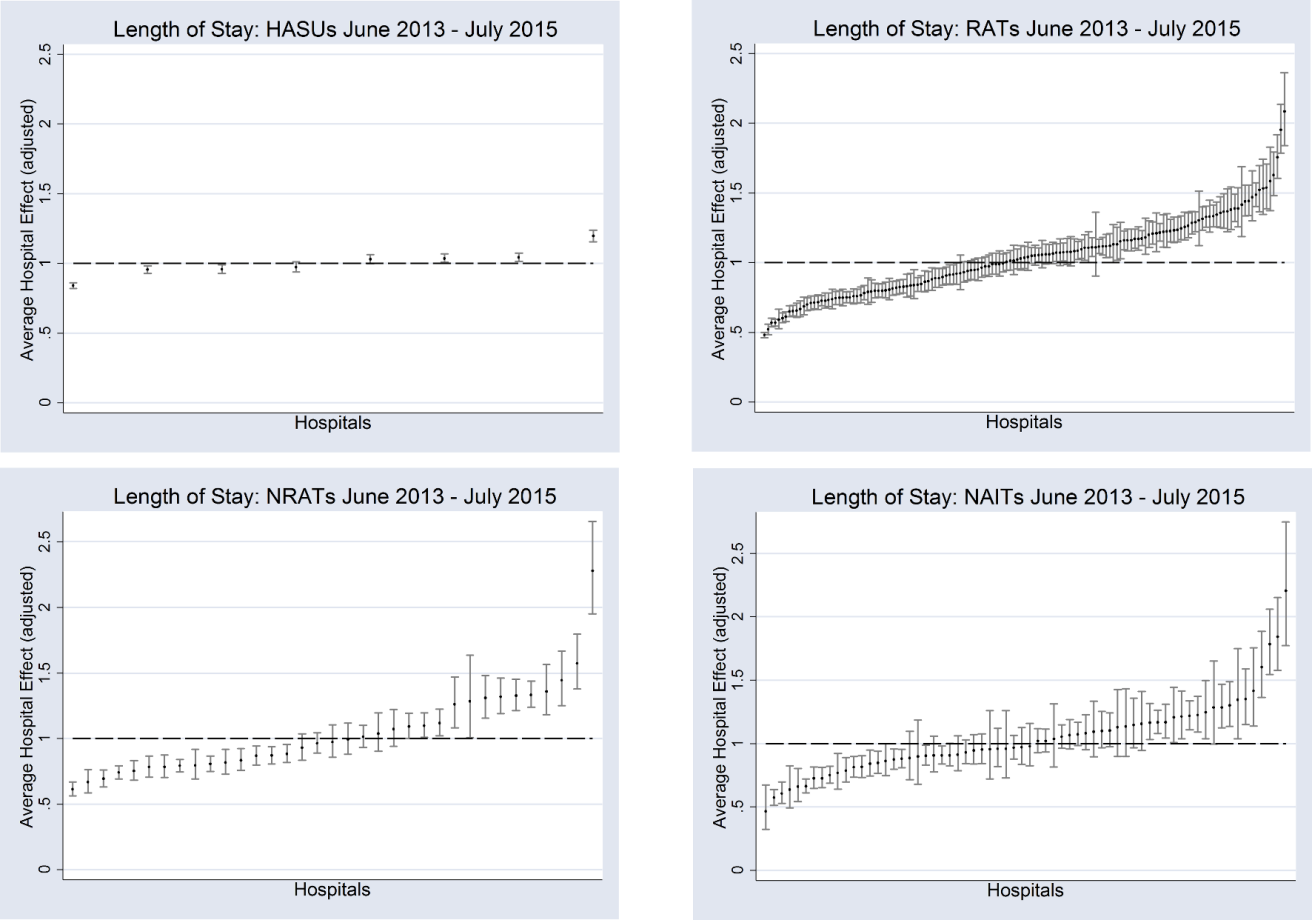
Unexplained variation in stroke team length of stay. HASUs, hyper-acute stroke units; NAITs, Non-Acute Inpatient Teams; NRATs, Non-Routinely admitting Acute Teams; RATs, Routinely Admitting Teams.

To ease the interpretation of stroke care team effects (purged from patient and admission-specific characteristics) as measures of relative performance, Figure 1 plots team effects standardised by the national average LoS for each team category.^10^ In this sense, a standardised team effect of 1.5 means that patients in the team in question have 50% longer LoS compared with the average for all teams in each category (not being due to the factors included in $X_{ik}$). Team effects are ranked by their deviation from the national average from left to right; those on the left-hand side have shorter LoS. It can be seen that even after conditioning on measurable patient and treatment characteristics there are large variations, within each team category, in the influence of stroke care teams on LoS. This difference is most noticeable amongst RATs (partly explained by the higher number of teams in this category) where the plot of standardised team effects follows an S-shaped distribution with clearly identified groups of teams having both significantly lower and significantly higher effect on LoS with respect to the national average. The average influence of the stroke team on LoS ranges from 19% below to 20% above the HASU national average; from 52% below to 107% above the RAT national average; from 39% below to 128% above the NRAT national average; and from 53% below to 121% above the NAIT national average.

The results of the second stage analysis are reported in table 4 (and online supplementary table S2—see online version). Lower team effects mean shorter than average LoS; consequently, a negative coefficient in the second stage can be interpreted as a positive association with stroke care performance. In general, the results of the second stage show that the variation in the performance measure remains highly unexplained as only few significant associations are found and the adjusted R^2^ in both cases is below 0.25.

**Table 4:** Stage 2: linear regression analysis—team effect on team factors

	RATs	NRATs
Stroke admissions (hundreds)	0.000	−0.019
	0.012	0.029
Mortality rate†	0.011	0.034**
	0.011	0.012
Rate of urinary infections†	0.014	0.005
	0.009	0.006
Rate of patients discharged as dependants†‡	−0.007**	−0.003
	0.003	0.015
Rate of WTE qualified clinical psychologists†	−0.404	−0.314
	0.979	1.066
Rate of WTE qualified dieticians†	0.901	0.299
	0.685	2.209
Rate of WTE qualified OT therapists†	−0.319	0.140
	0.332	3.506
Rate of WTE qualified PT therapists†	0.615	−0.188
	0.374	0.954
Rate of WTE qualified SALT therapists†	−0.048	0.601
	0.636	4.097
Rate of WTE registered nurses†	−0.012	−0.009
	0.079	0.067
Thrombolysis provided on site	−0.292*	0.055
	0.159	0.292
Access to stroke specific ESD	−0.098*	0.005
	0.055	0.258
Access to a non-specialist ESD	−0.029	0.072
	0.060	0.172
Access to non-specialist community rehabilitation team	−0.022	0.042
	0.065	0.261
Constant	1.528***	0.964
	0.354	1.450
N	147	32
R^2	0.327	0.566
Adjusted R^2	0.182	0.209

RATs model includes regional dummy variables; full model available online.

Coefficients; standard errors in second row, *p<0.10, **p<0.05, ***p<0.01.

†Rate per 100 hospitalisations.

‡Classified as dependant if mRS at final discharge >2.

ESD, Early supported discharge; mRS, modified Rankin Scale; NRATs, Non-Routinely admitting Acute Teams; OT, occupational therapy; PT, physiotherapy; RATs, Routinely Admitting Teams; SALT, speech and language therapy; WTE, whole time equivalent.

## Discussion

By estimating four separate multilevel count data models using length of inpatient stay (LoS) in the stroke team as the dependent variable and conditioning for patient and treatment characteristics, this analysis identifies the main drivers of resource use in stroke care, estimates stroke care team effects on the LoS of its patients, interpret these team effects as a performance measure and then estimates two linear models to analyse the variation in stroke care performance across two categories of stroke care teams.

The results of the multilevel models show that the main driver of LoS is the need for stroke therapy even after conditioning on stroke severity measured using both the mRS and the NIHSS scores. All the associations found were significantly different from zero at the 1% significance level and the magnitudes range between 34% longer LoS for stroke patients needing SALT in NAITs and 132% longer LoS for stroke patients needing PSY in RATs (each compared with patients not needing these therapies).

Interestingly, after conditioning for therapy needs and with the only exception of PT in NAITs, the amount of therapy received is positively associated with shorter inpatient stays at the 1% significance level for all kinds of therapy in all stroke care team categories. Since this finding is not showing a causal relationship, it should be interpreted with caution. However, this result should motivate future research into the overall clinical and economic consequences of improving early detection of therapy needs and increasing the amount of therapy provided to those stroke patients in need.

Results from the first stage model also show that dying in hospital is associated with 42% longer LoS in HASUs, but with significantly lower LoS in other team categories. These could be explained because HASUs were designed to provide hyper-acute care (rapid assessment with specialist clinical teams, diagnostic facilities and the capacity to provide thrombolysis) and to either discharge the patient or transfer him/her to a stroke team near his/her residence within 72 hours unless the patient is too unwell to be moved.^6 30^ Hence, these findings suggest that, on average, the poor condition of patients dying in HASUs prevented their transfer to a non-HASU team and, thus, stayed in the HASU longer than those well enough to be either discharged or transferred out. On the other hand, non-HASU teams have less pressure to discharge and/or transfer patients out than HASUs and, consequently, a patient will remain under the care of the stroke team until he/she is stable enough to be discharged or transferred out; his/her own death will shorten his/her stay.

Results from Stage 2 suggest that the rate of patients discharged as dependants is positively associated with RATs performance, but this association is not statistically significant for NRATs. By design, as briefly mentioned in the Introduction section, RATs typically provide acute stroke care over the first 7 days after stroke—table 1 shows an average LoS of 13.9 days. Depending on the patient’s progress, he/she will be discharged home or, if further inpatient care is needed, he/she will be transferred to an NRAT or a NAIT (both options are considered discharges from the RATs perspective). Once admitted to an NRAT or a NAIT, patients receive further care and rehabilitation until they are well enough to be discharged, thus tending to have longer stays than in other teams (average LoS of 23.1 and 37.4 days in NRATs and NAITs, respectively). Therefore, a possible explanation for the positive association between RATs performance and patient’s dependency rate is that, in general, RATs are doing a good job in timely identifying patients needing further inpatient care (likely to be in a dependant state) and in opportunely transferring them to NRATs or NAITs. Identifying different stroke care pathways and analysing their outcomes in terms of length of stay and dependency rates is needed to confirm this hypothesis. This is, however, beyond the scope of this paper.

As indicated by the adjusted deviance R^2^, proposed by Cameron & Windmeijer as a measure of goodness of fit for count data models and reported in table 2, the patient-level (Stage 1) analysis explains between 33% (NAITs) and 48% (RATs) of the variation in stroke LoS.^31^ A similar study analysing stroke LoS in England during 2007/08 explained up to 33% of the variation in LoS.^12^ One explanation for the better fit of the models estimated in the present paper could be the use of the SSNAP data that reports clinical information at the stroke admission level (eg, mRS and NIHSS scores, type and amount of therapy received at each stroke unit, etc.) which is not commonly recorded in the most commonly used administrative databases in the UK.

Despite recent re-organisation of stroke care services in the UK, there are important variations in the performance of stroke care teams, within each team category. These variations are not explained by measurable patient-related, admission-specific or stroke team characteristics (available in SSNAP) in any stroke team category. Therefore, these findings deserve further operational and financial analyses that can help unmask the cause of the huge variations in stroke care performance.

The analysis presented in this paper is subject to two main limitations. First, this study does not analyse costs variation across stroke teams since SSNAP does not provide cost information. A previous study using European data found that stroke costs and inpatient LoS are highly correlated^13^; however, a study using English data found that this does not necessarily apply for this country.^12^ The latter used data from 2007/08, that is, before the re-organisation of stroke care in England, Wales and Northern Ireland in 2010 and, therefore, it was not possible to consider the classification of stroke care teams in different categories which is acknowledged in the present study. Linking SSNAP with other databases reporting cost data at the individual level would allow complementing the present analysis with a similar one using patient and treatment costs as the outcome variables to explain resource use. This would represent an opportunity not only to explore if the relationship between costs and LoS has changed over time (compared with the Gaughan *et al* 12 study), but also if this relationship varies across stroke team categories.

The second main limitation of this analysis is that, given that SSNAP only includes stroke admissions, further hospital admissions with different primary diagnosis, as well as outpatient specialist and general practice visits following discharge from a stroke unit are not taken into account in this study. Consequently, the analysis conducted here is restricted to analysing resource use at the stroke team level and, as such, is only a partial resource use analysis. Future studies should exploit health care utilisation information included in other administrative datasets not readily linked to SSNAP (eg, Hospital Episodes Statistics, Clinical Practice Research Datalink, etc.) to analyse the extent to which reductions in the use of resources in the stroke unit is associated with overall health care resource use by stroke patients.
