# Supplementary material for: Variations in hospital resource use across stroke care teams in England, Wales and Northern Ireland: a retrospective observational study
Source: BMJ Open. 2019 Sep 20;9(9):e030426. doi: 10.1136/bmjopen-2019-030426 (PMC6756417; doi:10.1136/bmjopen-2019-030426)
Supplement: Supplementary file 1 [file bmjopen-2019-030426supp001.pdf]

Table S1. Results Stage 1: Negative binomial models for length of stay per team category

	HASUs (1)	RATs (2)	NRATs (3)	NAITs (4)
Age group, ref. value: 80-89				
age_lessthan50	1.034	0.693***	0.838***	0.900**
	0.027	0.012	0.035	0.047
age_50_59	0.989	0.706***	0.894***	0.892***
	0.021	0.010	0.030	0.031
age_60_69	1.031	0.770***	0.943**	0.961
	0.019	0.008	0.026	0.025
age_70_79	0.998	0.890***	0.961*	0.950***
	0.015	0.007	0.020	0.019
age_90_99	0.996	1.022**	0.950*	0.924***
	0.021	0.010	0.025	0.023
age_100 or more	0.952	0.901**	0.712***	0.784
	0.062	0.046	0.075	0.178
male	1.003	0.967***	1.027	0.981
	0.012	0.006	0.017	0.015
Ethnicity ref. value: white				
Asian	0.965*	0.993	1.007	0.964
	0.020	0.025	0.030	0.054
black	0.993	1.094*	1.116***	1.112
	0.023	0.052	0.042	0.091
mixed	1.109	1.114	1.091	1.148
	0.074	0.078	0.086	0.166
not known	0.993	0.980	0.987	0.998
	0.019	0.016	0.026	0.036
other	1.006	1.038	0.987	1.051
	0.021	0.050	0.036	0.069
Social deprivation ref. value: 1: low deprivation				
moderate deprivation	1.019	0.991	0.987	0.996
	0.015	0.010	0.022	0.025
high deprivation	0.966**	0.969***	1.029	0.989
	0.017	0.010	0.027	0.025

very high deprivation	0.964**	0.960***	1.030	1.003
	0.018	0.010	0.029	0.027
deprivation not known	1.033	0.948**	0.989	0.854**
	0.035	0.023	0.051	0.057
Stroke related co-morbidities				
heart failure	1.041*	1.007	0.967	0.973
	0.024	0.013	0.030	0.034
hypertension	1.008	1.003	1.009	1.017
	0.012	0.006	0.017	0.016
atrial fibrillation	0.997	1.035***	1.037*	1.007
	0.016	0.008	0.021	0.019
diabetes	1.006	1.048***	0.999	1.015
	0.014	0.008	0.019	0.021
previous stroke	0.985	0.977***	0.977	1.011
	0.013	0.007	0.018	0.018
Previous mRS score, ref. group: mRS=2				
previous MRS = 0 or previous MRS= 1	.	.	1.012	1.096*
	.	.	0.043	0.052
previous MRS = 3	.	.	1.145***	1.121***
	.	.	0.038	0.040
previous MRS = 4	.	.	1.467***	1.337***
	.	.	0.047	0.043
previous MRS = 5	.	.	1.655***	1.620***
	.	.	0.061	0.059
Severity of stroke, ref. group: mild (NIHSS < 5)				
moderate (NIHSS 5-14)	1.132***	1.533***	1.206***	1.184***
	0.015	0.012	0.026	0.024
severe (NIHSS 15-20)	1.189***	1.823***	1.402***	1.276***
	0.026	0.021	0.041	0.033
very severe (NIHSS > 20)	1.221***	1.838***	1.427***	1.292***
	0.029	0.022	0.047	0.037
intracerebral haemorrhage	1.081***	1.195***	1.098***	1.032
	0.022	0.012	0.025	0.022

Need for therapy				
need for occupational therapy (n_OT)	1.779***	2.306***	2.083***	1.695***
	0.040	0.029	0.078	0.114
need for physiotherapy (n_PT)	1.762***	1.754***	1.720***	1.691***
	0.046	0.025	0.075	0.115
need for speech and language therapy (n_SALT)	1.456***	2.121***	1.778***	1.336***
	0.025	0.019	0.040	0.026
need for psychological therapy (n_PSY)	1.613***	2.315***	1.920***	1.395***
	0.066	0.038	0.049	0.037
Amount of therapy				
n_OT x OT average daily minutes	0.985***	0.977***	0.986***	0.986***
	0.000	0.000	0.001	0.001
n_PT x PT average daily minutes	0.988***	0.995***	0.996***	1.001
	0.001	0.000	0.001	0.001
n_SALT x SALT average daily minutes	0.986***	0.970***	0.983***	0.994***
	0.001	0.001	0.001	0.001
n_PSY x PSY average daily minutes	0.986***	0.951***	0.973***	0.977***
	0.002	0.004	0.003	0.006
order of team in patient pathway	.	1.381***	1.057***	1.059*
	.	0.027	0.020	0.032
weekend admission	1.007	1.015**	1.029	1.008
	0.013	0.007	0.018	0.018
transferred from a HASU to another inpatient stroke unit	1.354	0.944	0.816***	0.758**
	0.262	0.071	0.020	0.086
Comorbidities				
patient treated for an urinary tract infection	1.122***	1.356***	1.179***	1.054*
	0.027	0.017	0.035	0.029
deceased	1.418***	0.830***	0.810***	0.765***
	0.049	0.009	0.024	0.044
transferred to another inpatient stroke unit	1.197***	1.070***	0.661***	0.670***
	0.017	0.011	0.016	0.031
constant	1.828***	2.512***	4.376***	8.768***
	0.051	0.066	0.291	0.940

N	14,720	112,339	11,693	6,644
alpha (dispersion)	0.128***	0.582***	0.494***	0.338***
adj. Deviance R^2	0.452	0.481	0.471	0.331

Exponentiated coefficients (IRR); Standard errors in second row. NB: negative binomial.

Team deviations not shown. * p<0.10, ** p<0.05, *** p<0.01

Table S2. Stage 2: Linear regression analysis - team effect on team factors

	RATs	NRATs
Stroke admissions (hundreds)	0.000	-0.019
	0.012	0.029
Mortality rate[ᵻ]	0.011	0.034**
	0.011	0.012
Rate of urinary infections[ᵻ]	0.014	0.005
	0.009	0.006
Rate of patients discharged as dependants[ᵻ,ǂ]	-0.007**	-0.003
	0.003	0.015
Rate of WTE qualified clinical psychologists[ᵻ]	-0.404	-0.314
	0.979	1.066
Rate of WTE qualified dieticians[ᵻ]	0.901	0.299
	0.685	2.209
Rate of WTE qualified OT therapists[ᵻ]	-0.319	0.140
	0.332	3.506
Rate of WTE qualified PT therapists[ᵻ]	0.615	-0.188
	0.374	0.954
Rate of WTE qualified SALT therapists[ᵻ]	-0.048	0.601
	0.636	4.097
Rate of WTE registered nurses[ᵻ]	-0.012	-0.009
	0.079	0.067
Thrombolysis provided on site	-0.292*	0.055
	0.159	0.292
Access to stroke specific ESD	-0.098*	0.005
	0.055	0.258
Access to a non-specialist ESD	-0.029	0.072
	0.060	0.172
Access to non-specialist community rehabilitation team	-0.022	0.042
	0.065	0.261
region, ref. group: Yorkshire and the Humber		
Cheshire and Mersey	0.013	.
	0.102	.
East Midlands	-0.300	.
	0.194	.
East of England	-0.003	.
	0.107	.
Greater MCR, Lancashire, South Cumbria	-0.234*	.
	0.134	.
North of England	-0.082	.
	0.098	.
Northern Ireland	-0.293	.
	0.312	.
South East Coast	-0.086	.
	0.099	.
South West	-0.095	.
	0.101	.
Thames Valley	-0.156	.

	0.176	.
Wales	-0.235	.
	0.165	.
Wessex	-0.032	.
	0.162	.
West Midlands	-0.230**	.
	0.108	.
_cons	1.528***	0.964
	0.354	1.450
N	147	32
R^2	0.327	0.566
adjusted R^2	0.182	0.209

Coefficients; standard errors in second row, * p<0.10, ** p<0.05, *** p<0.01
[†]Rate per 100 hospitalisations; [‡]classified as dependant if mRS at final discharge > 2.
